# Manganese-doped nano-hydroxyapatite enhances CF/PEEK osseointegration via immunomodulation-osteogenesis coupling

**DOI:** 10.1093/rb/rbag085

**Published:** 2026-05-04

**Authors:** Jiajun Liu, Zhenghao Li, Xuening Chen, Kai Zhang, Xiangdong Zhu, Bo Yuan

**Affiliations:** National Engineering Research Center for Biomaterials, Sichuan University, Chengdu 610064, China; College of Biomedical Engineering, Sichuan University, Chengdu 610064, China; National Engineering Research Center for Biomaterials, Sichuan University, Chengdu 610064, China; College of Biomedical Engineering, Sichuan University, Chengdu 610064, China; National Engineering Research Center for Biomaterials, Sichuan University, Chengdu 610064, China; College of Biomedical Engineering, Sichuan University, Chengdu 610064, China; National Engineering Research Center for Biomaterials, Sichuan University, Chengdu 610064, China; College of Biomedical Engineering, Sichuan University, Chengdu 610064, China; National Engineering Research Center for Biomaterials, Sichuan University, Chengdu 610064, China; College of Biomedical Engineering, Sichuan University, Chengdu 610064, China; National Engineering Research Center for Biomaterials, Sichuan University, Chengdu 610064, China; College of Biomedical Engineering, Sichuan University, Chengdu 610064, China

**Keywords:** CF/PEEK, Mn/nHA coating, immunomodulation, osteogenesis, osseointegration

## Abstract

Carbon fiber-reinforced polyetheretherketone (CF/PEEK) exhibits great application potential in repairing bone defects, particularly in large segmental defects at load-bearing sites, due to its advantages of bone-matching mechanical properties and radiolucency. However, its inherent bioinertness tends to induce interfacial fibrosis around the implant, leading to implant loosening or even failure. Herein, we employed a liquid-phase nanoparticle self-assembly strategy to deposit a manganese-doped nano-hydroxyapatite (Mn/nHA) coating *in situ* on the surface of CF/PEEK, aiming to synergistically enhance the immunomodulatory capacity and bioactivity. *In vitro* co-culture experiments demonstrated that the Mn/nHA coating effectively induced the polarization of macrophages toward the M2 anti-inflammatory phenotype, inhibited the secretion of pro-inflammatory factors and created an immune microenvironment conducive to tissue repair. Meanwhile, it promoted the proliferation and osteogenic differentiation of BMSCs. *In vivo* femoral condyle defect repair experiments further confirmed that the Mn/nHA functional coating significantly inhibited interfacial fibrosis around the CF/PEEK and accelerated bone tissue regeneration, and interfacial osseointegration through an immunomodulatory-osteogenic coupling effect. This study provides new strategies and references for optimizing the bioactivity of CF/PEEK, addressing interfacial compatibility challenges and designing implant materials for bone defect repair.

## Graphical abstract

**Graphical Abstract:**
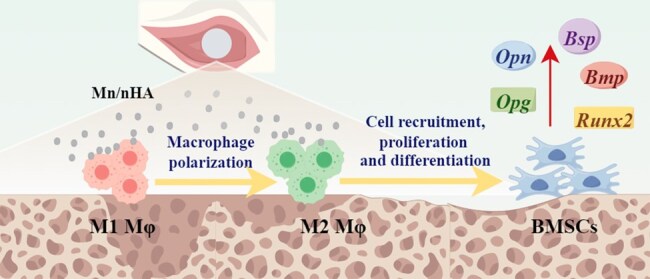

## Introduction

Repairing large segmental bone defects in load-bearing areas and achieving functional reconstruction remain a significant and long-standing clinical challenge in orthopedics, as such defects rarely heal spontaneously and typically require intervention with bone graft materials [1–3]. Medical metals with excellent mechanical properties and biocompatibility hold a dominant position among clinical materials for repairing large segmental bone defects, while they present issues such as excessively high modulus, corrosion leading to metal ion release and imaging artifacts that interfere with radiological examination [4]. Carbon fiber-reinforced polyetheretherketone (CF/PEEK; abbreviated as C/P below) composites, characterized by an elastic modulus matching natural human bone, superior mechanical properties and radiolucency, have gradually emerged as ideal implant materials to replace medical metals [5]. Nevertheless, the inherent bioinertness of C/P often leads to the formation of extensive fibrous tissue at the implant interface, creating a fibrous encapsulation. Under functional loading, this can result in loosening or even failure of the implant, severely compromising the long-term stability and clinical repair efficacy of C/P [6, 7].

The essence of implant interface fibrosis lies in excessive immune regulation by the body [8]. On the surface of bioinert material, immune cell activation occurs, followed by a polarization shift toward a sustained pro-inflammatory state [9]. The subsequent secretion of cytokines and growth factors drives the over-activation of fibroblasts, leading to fibrous encapsulation [9, 10]. A viable strategy involves guiding the immune response toward a pro-repair, pro-regenerative, mild inflammatory state by modulating the surface properties of implants [11–14]. For example, promoting macrophage polarization toward the M2 phenotype, known for its anti-inflammatory and pro-healing functions, can lead to the secretion of signaling molecules favorable for angiogenesis and osteogenic differentiation [15, 16]. Through such immunomodulation, the unfavorable fibrotic process can be fundamentally suppressed, while simultaneously creating a microenvironment conducive to the migration, proliferation and osteogenic differentiation of mesenchymal stem cells [17–19]. Thus, endowing C/P materials with immunomodulatory capabilities might be a feasible approach to achieve rapid interfacial osseointegration.

In addition to immunomodulation, researchers have also extensively explored the strategy of directly conferring bioactivity to materials to enhance their bone regeneration potential [20]. Nano-hydroxyapatite (nHA), as the primary inorganic component of bone tissue, possesses good biocompatibility and unique nano-effects, and is extensively used as a nanocarrier or coating material in bone tissue engineering and regenerative repair [21]. Although most current research focuses on the osteogenic properties, some studies have found that nHA also exhibits certain immunomodulatory abilities, which can enhance its role in tissue regeneration [22–24]. For example, manganese (Mn), an essential trace mineral in the human body, plays a role in regulating bone metabolism and maintaining skeletal structure [25]. More importantly, extracellular Mn ions can activate immune responses in macrophages, dendritic cells and lymphocytes via the circulatory system [26]. Once entering the cytoplasm, Mn ions can strongly activate the cGAS-STING pathway, significantly enhancing the synthesis of cyclic GMP-AMP (cGAMP) [27].

Therefore, many studies have attempted to enhance immunomodulatory capacity through Mn-doped HA, but this approach has mainly been used to regulate the biological effects of tumor cells, with little focus on enhancing bone regeneration [26–29]. Moreover, due to the need for tumor cell killing, the morphology of Mn-doped HA is mostly needlelike, which also exerts a cytotoxic effect on normal osteoblasts [30–33].

**Figure 1:**
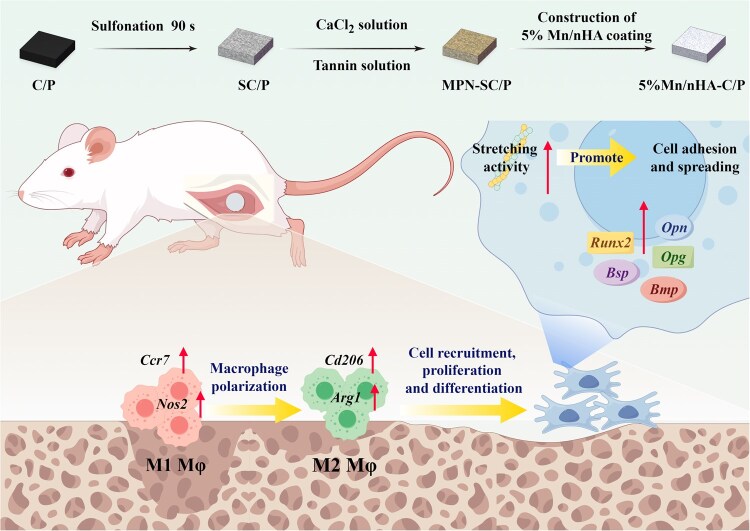
Mechanism of immunomodulation-osteogenesis coupling and osseointegration enhancement of CF/PEEK modified by Mn/nHA.

In this study, we constructed a granular Mn/nHA coating on the surface of C/P composites through a liquid-phase nanoparticle self-assembly strategy, aiming to enhance its bioactivity and immunomodulatory capacity for addressing the challenge of interfacial fibrosis (Figure 1). Firstly, C/P was pretreated via sulfonation to introduce hydrophilic functional groups and a porous micro-network on its surface. Simultaneously, mimicking mussel adhesion mechanisms, a metal-phenolic network (MPN) was constructed on the sulfonated C/P surface to provide active sites for the subsequent *in situ* nucleation and growth of nHA. *In vitro* co-culture models involving BMSCs and macrophages were established to evaluate the osteogenic and immunomodulatory capabilities of different C/P composites. Finally, a rat femoral condyle defect model was created to assess the *in vivo* osseointegration ability of C/P implant.

## Materials and methods

### Sample preparation

Medical-grade C/P plates were cut into square samples with dimensions of 8 mm × 8 mm × 2 mm. After fine polishing with 800-mesh silicon carbide sandpaper, the samples were ultrasonically cleaned in acetone, absolute ethanol and ultrapure water for 15 min each in sequence, then dried in a clean environment for later use. The pretreated samples were immersed in 98% concentrated sulfuric acid for sulfonation for 90 s, quickly transferred to ultrapure water for overnight soaking and dried to obtain sulfonated C/P (SC/P). The preparation steps of the intermediate layer are as follows: first, a 1.21 mg/mL Tris solution was prepared, and then a 12 mg/mL anhydrous calcium chloride solution and a 6 mg/mL tannic acid solution were prepared using this Tris solution as the base; at room temperature, SC/P was immersed in the tannic acid solution, fully stirred for infiltration, then anhydrous calcium chloride solution was quickly added. The mixture was placed in an oven at 37°C for overnight deposition, finally rinsed repeatedly with ultrapure water and dried at 37°C for later use. For the construction of the nHA bioactive coating, pretreated C/P was added to the nHA synthesis system to achieve *in situ* growth of nHA, with simultaneous doping of 5% Mn element according to previous literature [45, 49]. The specific steps are as follows: prepare the Ca solution (0.5 mol/L Ca(NO_3_)_2_·4H_2_O), Ca/Mn mixed solution (0.45 mol/L Ca(NO_3_)_2_·4H_2_O + 0.05 mol/L Mn(NO_3_)_2_·6H_2_O) and P solution (0.3 mol/L (NH_4_)_2_HPO_4_), ensuring the molar ratios of Ca/P and (Ca + Mn)/P are both 5:3; immerse the C/P loaded with MPN intermediate layer into the above two Ca-based solutions respectively, soak for 30 min until fully infiltrated; slowly add the P solution dropwise to the system, adjust the pH to approximately 10.0 with ammonia water under room temperature stirring to promote nHA nucleation; after the dropwise addition of P solution is completed, continue stirring for 30 min, then stand for aging for 24 h to facilitate uniform deposition and crystalline stability of the coating; finally, take out the materials, rinse thoroughly with deionized water and dry in a clean environment. Two composite materials were obtained: Mn-untreated nHA-coated C/P (nHA-C/P) and 5% Mn-doped nHA-coated C/P (5% Mn/nHA-C/P). The fabrication process of the material and its mediated osteointegration enhancement mechanism in this study are illustrated in Figure 1, which was drawn by figdraw.com.

### Material physicochemical characterization

Scanning electron microscopy (SEM) was employed to observe the surface morphology of carbon fiber/PEEK (C/P) composites under different sulfonation times (60 s, 90 s, 120 s). Additionally, FE-SEM characterization was performed on pristine C/P, SC/P treated with the optimal sulfonation time and MPN-SC/P with a deposited MPN intermediate layer. Meanwhile, EDS attached to FE-SEM was used for preliminary analysis of the elemental composition and investigation of distribution uniformity of pure nHA powder and 5% Mn/nHA powder, aiming to explore the synthesis conditions of Mn-doped hydroxyapatite (HA) powder. Fourier transform infrared spectroscopy (FTIR) was utilized to analyze the differences in chemical groups between the two groups of nanoparticles, X-ray diffraction (XRD) was used to characterize their phase compositions, and XPS was employed for elemental valence state analysis and semi-quantitative composition analysis. To evaluate the release behavior of particles from the coating surface, three groups of samples (C/P, nHA-C/P, and 5% Mn/nHA-C/P) were separately immersed in Tris buffer solution (pH = 7.4) and incubated in an oven at 37°C for 1, 4 and 7 days. The solutions were collected at each time point, and inductively coupled plasma atomic emission spectroscopy (ICP-AES) was used for quantitative determination of Mn ion concentration. Three parallel samples were set for each test point. Transmission electron microscopy (TEM) was used to observe the particle size and morphology of nanoparticles in the surface coatings of nHA-C/P and 5% Mn/nHA-C/P, and the doping status of Mn in nHA was analyzed by EDS attached to TEM to investigate the microcharacteristics of the nanoparticles in the coatings.

### Evaluation of *in vitro* biological properties of C/P composites modified with Mn/nHA

#### Sample preparation and sterilization

Three groups of samples (C/P, nHA-C/P and 5% Mn/nHA-C/P) with dimensions of 8 mm × 8 mm × 2 mm were placed in Petri dishes, sealed with sterilization indicator tape and sterilized by autoclaving at 121°C for 30 min. After sterilization, the samples were dried in an oven. Prior to use, the Petri dishes were disinfected with 75% alcohol spray, and the materials were transferred to a laminar flow hood for subsequent experiments.

#### Isolation and culture of rBMSCs

Rat BMSCs were cultured in α-MEM medium supplemented with 10% fetal bovine serum (FBS) and 1% penicillin/streptomycin, under standard conditions (37°C, 5% CO_2_). The culture medium was refreshed every 48 h. When cell confluency reached 80–90%, the cells were rinsed 2–3 times with sterile phosphate buffered saline (PBS) to remove residual medium. Digestion was initiated by adding 1 mL of trypsin, and monitored under an optical microscope. Once cell edges became refractile and morphology rounded, fresh complete medium was added to terminate digestion. The cell suspension was gently pipetted to achieve a homogeneous mixture, and subcultured at a 1:3 split ratio. Cells from passages 3–5, exhibiting stable growth, were selected for subsequent experiments.

#### Evaluation of BMSC cytocompatibility and adhesion

The cytocompatibility of the three sample groups was assessed using FDA/PI live-dead staining. Rhodamine-labeled phalloidin (for cytoskeleton visualization) and DAPI (for nuclear staining) were used in conjunction with CLSM to observe the spreading morphology and adhesion of BMSCs on the sample surfaces. Following gradient dehydration, critical point drying and gold sputtering, the samples were imaged via SEM to examine the microscale spreading and adhesion of cells.

#### Assessment of osteogenic differentiation

RT-qPCR was employed to quantify the expression levels of osteogenesis-related genes after 7 days of co-culture between BMSCs and the three sample groups. ALP staining was performed to evaluate the osteogenic differentiation potential of BMSCs.

#### Culture of mouse macrophages

Mouse macrophage cell line RAW264.7 was cultured in high-glucose DMEM containing 10% FBS and 1% penicillin–streptomycin, under conditions of 37°C and 5% CO_2_ with saturated humidity. When adherent cells reached 70–80% confluency, they were gently washed twice with PBS, resuspended in fresh complete medium and subcultured at a 1:3 ratio. Log-phase cells with a round, granular morphology were selected for further experiments.

#### Evaluation of macrophage polarization

After 7 days of co-culture with the materials, immunofluorescence staining combined with CLSM was used to detect the expression and localization of the M1 marker iNOS and the M2 marker CD206 in RAW264.7 cells. The polarization state was assessed by quantitative analysis of fluorescence intensity. RT-qPCR was performed to quantify the mRNA expression levels of macrophage polarization-related genes, including *Nos2*, *Ccr7*, *Cd206* and *Arg1* at 4 and 7 days post co-culture.

### Evaluation of *in vivo* osteogenic performance of C/P composites modified with Mn/nHA

#### Sample preparation and sterilization

During the experimental preparation phase, three types of materials (C/P, nHA-C/P and 5% Mn/nHA-C/P) were processed into cylindrical samples with a diameter of 3 mm and a height of 3.5 mm. The samples were placed in clean glass Petri dishes, sealed with medical-grade sterilization indicator tape and subjected to high-pressure steam sterilization at 121°C and 0.1 MPa for 30 min. After sterilization, the samples were stored in a laminar flow hood and used for animal implantation surgery within 24 h to ensure sterility.

#### Animal selection and bone defect model establishment

Eight-week-old healthy female Sprague-Dawley (SD) rats, with an average body weight of 180 ± 15 g, were provided by the Experimental Animal Center of Sichuan University. All experimental procedures were performed in compliance with the approved protocol of the Ethics Committee of the aforementioned center (Ethics approval number: KS2023327). A critical-sized bone defect (CSBD) model was established in the femoral condyle of rats, with the defect designed as 3.0 ± 0.1 mm in diameter and 4.0 ± 0.2 mm in depth at the central bone surface, to evaluate the *in vivo* rapid osseointegration capacity of the three groups of materials.

#### Sample collection and detection analysis

At 2 weeks and 4 weeks after material implantation, rat blood was collected for routine blood tests, as well as the determination of serum Mn^2+^ and Ca^2+^ concentrations. High-resolution µCT (Scanco Medical AG, Bassersdorf, Switzerland) was employed for 3D morphological analysis of bone tissue specimens. Subsequent to gradient dehydration and paraffin embedding, the bone tissue specimens were sectioned into 5–7 μm continuous slices using a microtome. After dewaxing to water and gradient rehydration, toluidine blue staining and methylene blue-basic fuchsin staining were conducted, respectively, for histological observation.

### Statistical analysis

Statistical significance was determined using one‑way analysis of variance (one‑way ANOVA) for comparisons among three groups at a single time point, and two‑way analysis of variance (two‑way ANOVA) for comparisons among three groups involving two or more time points. *P *< 0.05 was considered statistically significant.

## Results

### Material preparation and characterization

**Figure 2:**
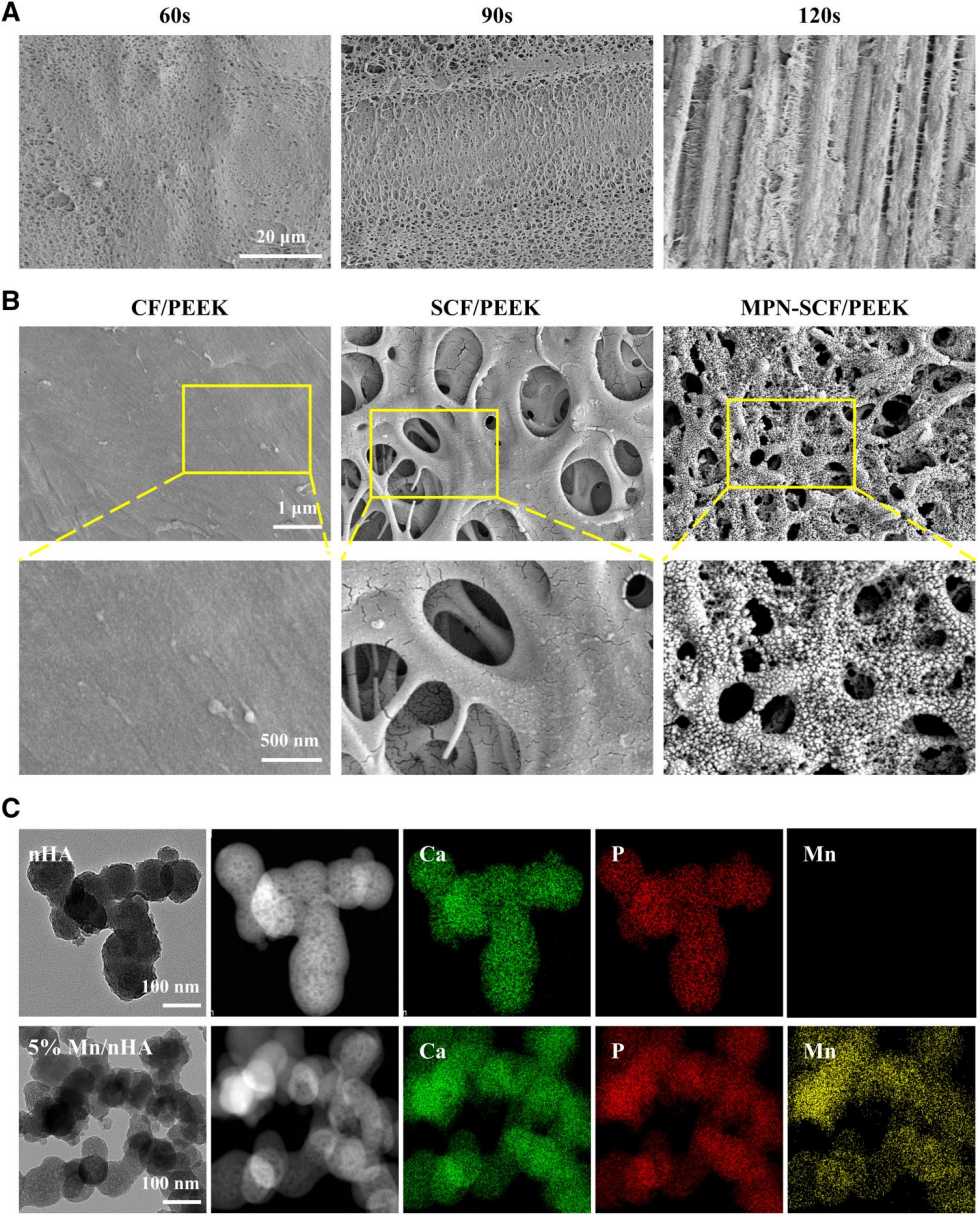
Characterization and identification of the material preparation process. (**A**) SEM images of C/P materials sulfonated for 60, 90 and 120 s. (**B**) SEM images of C/P, SC/P after 90 s sulfonation and MPN-SC/P modified with MPN; (**C**) TEM images and EDS mapping images of nHA and 5% Mn/nHA.

For brevity, pristine CF/PEEK was designated as C/P, sulfonated CF/PEEK as SC/P, MPN-coated SC/P as MPN‑SC/P and MPN‑SC/P loaded with nano‑HA and 5 mol% Mn‑doped nano‑HA as nHA‑SC/P and 5%Mn/nHA‑SC/P, respectively. SEM analysis of the effect of different sulfonation times on C/P (Figure 2A) showed that sulfonation for 120 s led to excessive exposure of carbon fibers, failing to provide effective bioactive sites; sulfonation for 60 s resulted in insufficient formation of micro–nano porous structures on the surface due to incomplete reaction. Therefore, 90 s was selected as the optimal sulfonation parameter in this study. Surface morphology analysis (Figure 2B) indicated that the unsulfonated C/P had a smooth and flat surface, with visible long strip-shaped carbon fibers and PEEK matrix; after sulfonation with concentrated sulfuric acid, a randomly distributed nano-to-micron scale porous structure was formed on the surface, which not only increased surface roughness but also expanded the specific surface area, providing potential attachment sites for subsequent nHA coating loading. Despite the porous structure of SC/P, the presence of carbon fibers compressed the sulfonation reaction time of PEEK, making it difficult to form sufficient nHA binding sites. Thus, an intermediate layer needs to be introduced on the SC/P surface to supplement binding sites [34]. In this study, tannic acid and anhydrous calcium chloride were used to prepare the MPN coating (Figure 2B). Nanoparticles were uniformly distributed on the material surface, inside the pores and within the network structure, which not only further improved surface roughness but also provided abundant nucleation sites for nHA growth through Ca^2+^ in MPN, ensuring effective nHA loading.

**Figure 3:**
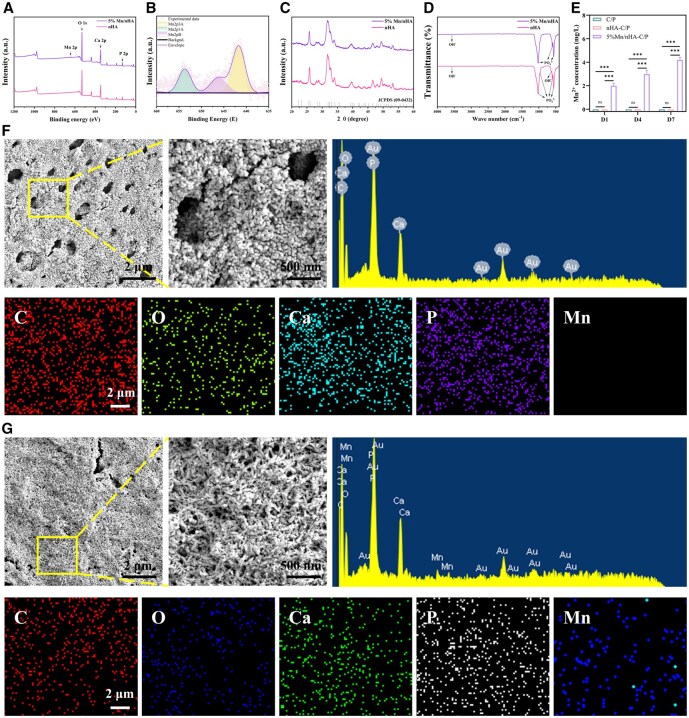
Characterization results of material preparation. (**A**) XPS full spectra of nHA and 5% Mn/nHA. (**B**) High-resolution XPS spectrum of 5% Mn/nHA. (**C**) XRD patterns of nHA and 5% Mn/nHA. (**D**) FTIR spectra of nHA and 5% Mn/nHA. (**E**) Mn ion release profiles of C/P, nHA-C/P and 5% Mn/nHA-C/P scaffolds determined by ICP. (**F**) SEM images and EDS characterization of the morphology and elemental composition of nanoparticles on the surface of nHA-C/P scaffolds. (**G**) SEM images and EDS characterization of the morphology and elemental composition of nanoparticles on the surface of 5% Mn/nHA-C/P scaffolds.

TEM was used to characterize the morphology of nanoparticles. As shown in Figure 2C, both types of nanoparticles were mainly granular with a particle size of approximately 50–100 nm and slight agglomeration. To clarify the doping status of Mn element, mapping analysis was performed using an energy-dispersive spectrometer (EDS) attached to TEM. The results showed that in addition to the signals of Ca and P elements, the Mn element signal in 5% Mn/nHA was significant and uniformly distributed, confirming that Mn had been successfully doped into the HA lattice. To verify whether Mn was successfully doped into nHA, X-ray photoelectron spectroscopy (XPS) was first employed for characterization (Figure 3A). In the XPS full spectra of the two nanoparticles, pure nHA only exhibited characteristic peaks of Ca and P elements, while the 5% Mn/nHA sample showed an additional distinct Mn 2p characteristic peak. Combined with the Mn 2p high-resolution XPS spectrum fitting results in Figure 3B, this not only directly confirms the successful introduction of Mn into the system but also indicates that Mn exists in multiple coordination environments within HA. Further high-resolution XPS spectrum fitting analysis of O 1s, P 2p and Ca 2p (Supplementary Figure S1A–C) revealed that the binding energies of these three elements in 5% Mn/nHA were identical to those in pure nHA, demonstrating that Mn doping did not alter the core electronic coordination environments of Ca, P and O. However, the full widths at half maximum (FWHMs) of O 1s and Ca 2p were slightly larger than those of pure nHA, reflecting minor inhomogeneity in the electronic environments of O and Ca on the sample surface after Mn doping. This effect may be ascribed to the localized presence of Mn–O bonds and the substitution of a small fraction of Ca sites by Mn. In contrast, the FWHM of P 2p showed no significant change.

Subsequently, XRD was used to analyze the phase compositions of nanoparticles. As shown in Figure 3C, the characteristic diffraction peaks of both nanoparticles were fully consistent with the typical peaks of the HA standard card (JCPDS: 09-0432). Notably, although both exhibited the HA characteristic diffraction peak around 31.8°, the peak of 5% Mn/nHA showed a slight high-angle shift. Since the atomic radius of Mn^2+^ is smaller than that of Ca^2+^, the substitution of partial Ca^2+^ causes lattice contraction and a decrease in lattice parameters of HA, thereby leading to a slight high-angle shift of the diffraction peak [35]. Finally, FTIR (Figure 3D) was used to analyze the chemical composition. Both spectra showed typical characteristic absorption peaks of nHA, including the stretching vibration peak of OH^−^ around 3445 cm^−1^, and the characteristic stretching vibration peaks of ${\mathrm{PO}}_{4}^{3-}$ around 560 cm^−1^, 600 cm^−1^ and 1020 cm^−1^ [36]. Further comparison indicated that the FTIR characteristic peaks of 5% Mn/nHA did not show significant wavenumber shifts, suggesting that Mn doping did not significantly change the vibration environments of the main functional groups of nHA. As presented in Figure 3E, no Mn^2+^ release was observed from the C/P and nHA‑C/P composites owing to the absence of Mn doping. In contrast, the 5% Mn/nHA‑C/P composite displayed a sustained release of Mn^2+^, with the ion concentration reaching 4.2 ± 0.22 mg/L at day 7.

The surface morphology and elemental composition of nHA-C/P and 5% Mn/nHA-C/P materials were characterized by SEM combined with EDS. As shown in Figure 3F and G, after surface modification with nHA or 5% Mn/nHA, significant nanoparticle deposition was observed on the surface of the C/P substrate. EDS elemental analysis results showed that C, O, Ca and P elements were detected on the surface of nHA-C/P, while no Mn element was found. In contrast, in addition to C, O, Ca and P elements, Mn element was clearly detected on the surface of 5% Mn/nHA-C/P, further confirming that Mn was successfully introduced onto the material surface along with the modified layer.

### *In vitro* immunomodulatory performance of 5% Mn/nHA-C/P

**Figure 4:**
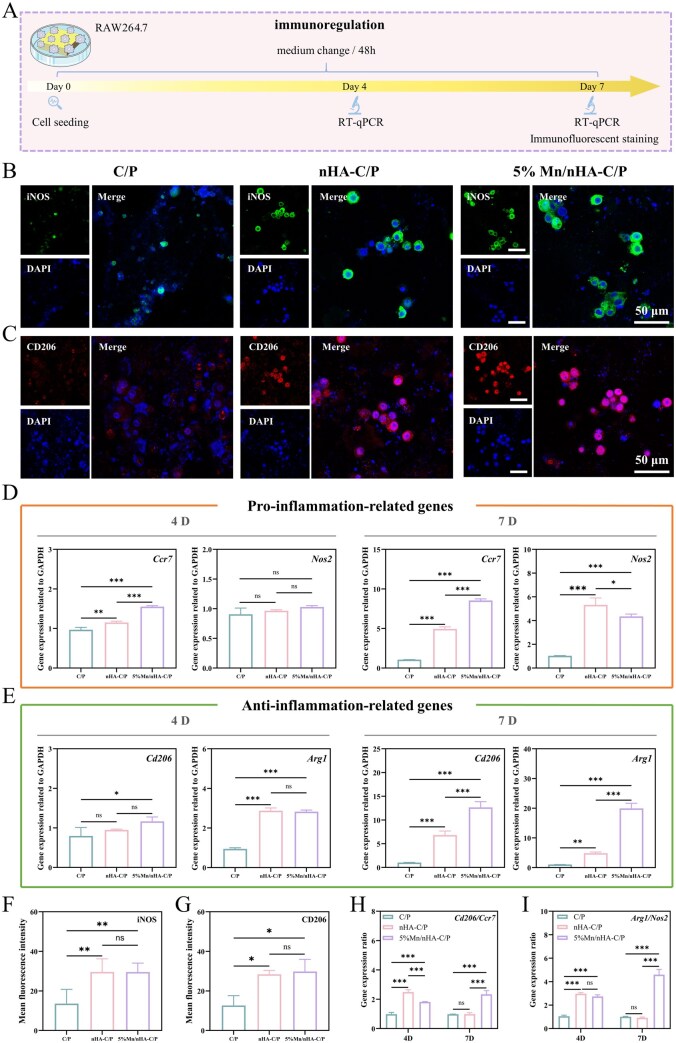
Characterization of the *in vitro* immunomodulatory properties. (**A**) Schematic diagram of RAW264.7 cell experiments. (**B**) iNOS immunofluorescence staining and (**F**) its semi-quantitative results of C/P, nHA-C/P and 5% Mn/nHA-C/P scaffolds co-cultured with RAW264.7 cells for 7 days; (**C**) CD206 immunofluorescence staining and (**G**) its semi-quantitative results of the three groups of materials co-cultured with RAW264.7 cells for 7 days. (**D**) RT-qPCR results for pro-inflammatory-related genes (*Ccr7* and *Nos2*) at 4 and 7 days. (**E**) RT-qPCR results for anti-inflammatory-related genes (*Cd206* and *Arg1*) at 4 and 7 days. (**H**) *Cd206*/*Ccr7* ratio at 4 and 7 days; (**I**) *Arg1*/*Nos2* ratio at 4 and 7 days. * refers to *P *< 0.05, ** refers to *P *< 0.01, *** refers to *P *< 0.001.

Figure 4A shows the schematic diagram of the *in vitro* immunomodulation experiment of the materials. Immunofluorescence staining results (Figure 4B and C) revealed that compared with the C/P group, the macrophages on the surfaces of the nHA-C/P group and 5% Mn/nHA-C/P group exhibited significant changes in the expression of M1-type macrophage marker inducible nitric oxide synthase (iNOS) and M2-type macrophage marker mannose receptor C type 1 (CD206). Semi-quantitative analysis of immunofluorescence revealed that both the nHA-C/P and 5% Mn/nHA-C/P groups exhibited extremely significant differences in iNOS intensity compared to the C/P group (*P *< 0.01), while their CD206 intensities also showed significant differences (*P *< 0.05) (Figure 4F and G).

To systematically elucidate the regulatory effects of different scaffold materials on macrophage polarization phenotype and inflammatory microenvironment, the expression levels of M2-type anti-inflammatory genes (*Cd206* and arginase 1 [*Arg1*]) and M1-type pro-inflammatory genes (C–C motif chemokine receptor 7 [*Ccr7*] and inducible nitric oxide synthase 2 [*Nos2*, *iNos*]) were detected via reverse transcription quantitative real-time polymerase chain reaction (RT-qPCR), and the M2/M1 polarization ratios (*Cd206*/*Ccr7*, *Arg1*/*Nos2*) were calculated.

Regarding the expression of M1-type pro-inflammatory-related genes (Figure 4D): On day 4 of culture (the early inflammatory stage), the expression level of *Ccr7* was significantly upregulated in both the nHA-C/P and 5% Mn/nHA-C/P groups compared with the C/P control group (*P* < 0.01), and the upregulation in the 5% Mn/nHA-C/P group was significantly greater than that in the nHA-C/P group. No significant difference in *Nos2* expression was observed among all the groups, although the 5% Mn/nHA-C/P group still showed a trend toward higher expression. By day 7, the expression of both *Ccr7* and *Nos2* was significantly higher in the nHA-C/P and 5% Mn/nHA-C/P groups than in the C/P group (*P* < 0.001), and *Nos2* expression in the 5% Mn/nHA-C/P group was slightly decreased compared with the nHA-C/P group.

Regarding the expression of M2-type anti-inflammatory genes (Figure 4E): On day 4, the expression levels of *Cd206* and *Arg1* in the nHA-C/P and 5% Mn/nHA-C/P groups were both higher than those in the C/P group, among which *Arg1* was significantly upregulated in both the modified groups (*P* < 0.001). By day 7, the expression of *Cd206* and *Arg1* in the nHA-C/P and 5% Mn/nHA-C/P groups was extremely significantly elevated compared with the C/P group (*P* < 0.001), and the expression levels of *Cd206* and *Arg1* in the 5% Mn/nHA-C/P group were significantly higher than those in the nHA-C/P group.

The M2/M1 gene ratio serves as a key indicator reflecting the direction of macrophage polarization and the status of the inflammatory microenvironment (Figure 4H and I). On day 4, the *Cd206*/*Ccr7* and *Arg1*/*iNos* ratios in both the nHA-C/P and 5% Mn/nHA-C/P groups were significantly higher than those in the C/P group (*P* < 0.001), with the ratios in the 5% Mn/nHA-C/P group being notably lower than those in the nHA-C/P group. By day 7, the *Cd206*/*Ccr7* and *Arg1*/*Nos2* ratios in the 5% Mn/nHA-C/P group increased markedly and surpassed those in the nHA-C/P group (*P* < 0.001). In contrast, the *Cd206*/*Ccr7* ratio in the nHA-C/P group was only slightly higher than that in the C/P group, and its *Arg1*/*Nos2* ratio showed no significant difference from the C/P group. This dynamic trend clearly demonstrates that the 5% Mn/nHA-C/P scaffold promotes a more rapid and thorough transition of macrophages from the pro-inflammatory M1 phenotype to the anti-inflammatory M2 phenotype. While activating the immune response in the early stage, it promptly shifts the inflammatory microenvironment toward a reparative state, thereby creating favorable conditions for osteogenic repair. In contrast, although the nHA-C/P scaffold can induce M2 polarization, its conversion efficiency and temporal regulatory capacity are significantly weaker than those of the Mn-doped group.

### *In vitro* osteogenic differentiation performance of 5% Mn/nHA-C/P

**Figure 5:**
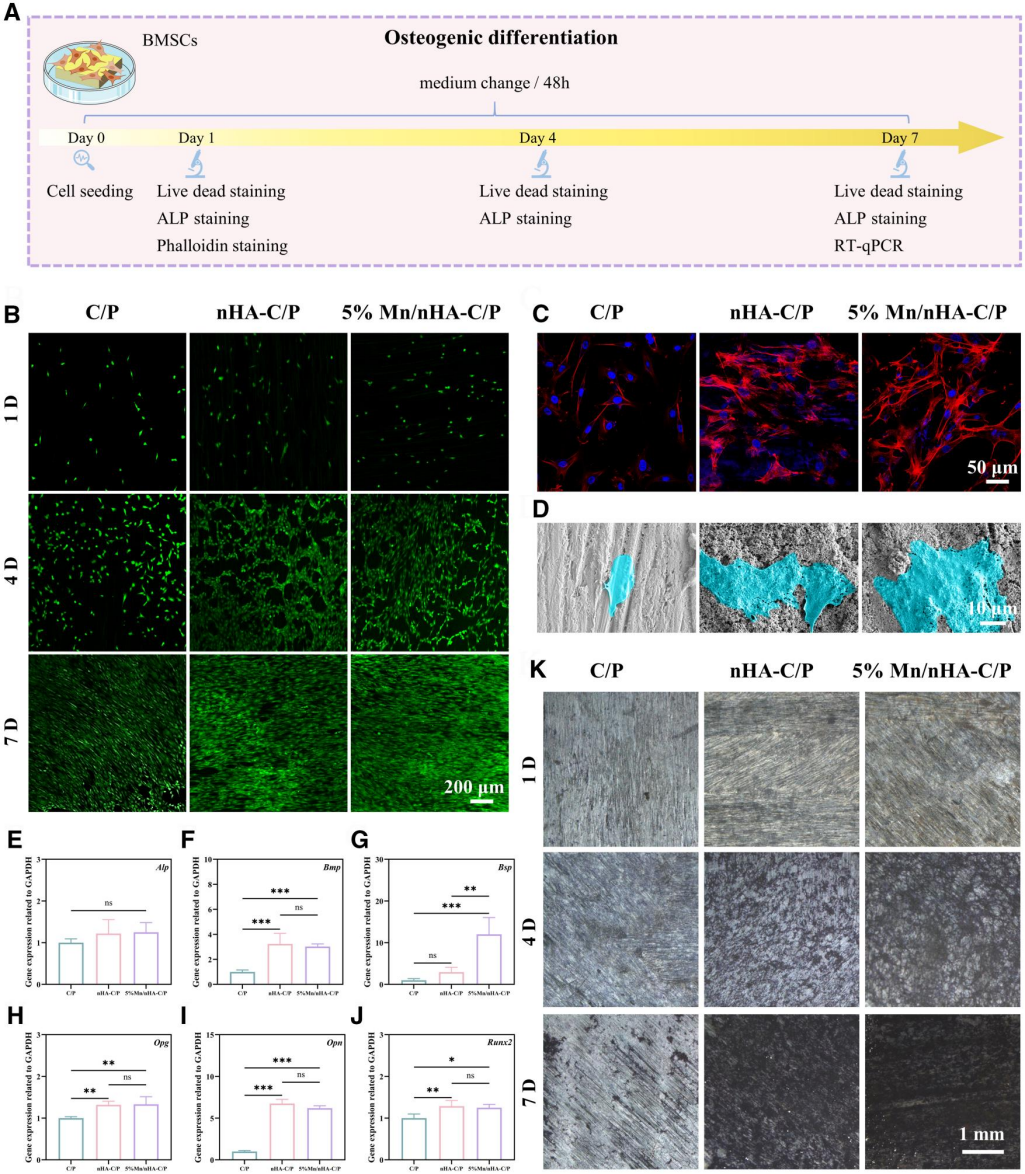
Characterization of the *in vitro* osteogenic properties. (**A**) Schematic diagram of BMSCs experiments. (**B**) Live/dead staining images of BMSCs co-cultured with C/P, nHA-C/P, and 5% Mn/nHA-C/P scaffolds for 1, 4 and 7 days. (**C**) CLSM images and (**D**) SEM images of phalloidin-stained BMSCs after 1 day of culture on the three groups of materials. (**E–J**) RT-qPCR results of (**E**) *Alp*, (**F**) *Bmp*, (**G**) *Bsp*, (**H**) *Opg*, (**I**) *Opn* and (**J**) *Runx2* after co-culturing BMSCs with the three groups of materials for 7 days. (**K**) ALP staining results of BMSCs cultured on the three groups of materials for 1, 4 and 7 days. * refers to *P *< 0.05, ** refers to *P *< 0.01, *** refers to *P *< 0.001.

Figure 5A presents the schematic diagram of the *in vitro* osteogenic differentiation-promoting effect of the materials. To evaluate the cytocompatibility, cell growth and proliferation of the three groups of materials, FDA/PI staining combined with confocal laser scanning microscopy (CLSM) was used to analyze the cell viability after 1, 4 and 7 days of culture (Figure 5B). The results showed that throughout the culture period, cells on the surfaces of different materials exhibited normal spreading morphology, and the cell density gradually increased with culture time, indicating that all three groups of materials possessed good cytocompatibility. To further investigate the cell spreading morphology, BMSCs were co-cultured with the materials for 1 day, followed by phalloidin staining for cytoskeleton and DAPI staining for cell nuclei, and observation by CLSM (Figure 5C). Few cells adhered to the C/P surface, with a small number of short filopodia and a spindle-shaped morphology. In contrast, the number of adherent cells on nHA-C/P and 5% Mn/nHA-C/P surfaces increased significantly, with more obvious cytoplasmic expansion, increased and longer filopodia and a more spread cell morphology.

We further observed the spreading and adhesion morphology of BMSCs during co-culture using SEM (Figure 5D). BMSCs could normally adhere and spread on the surfaces of all three groups of materials, but the cells on nHA-C/P and 5% Mn/nHA-C/P surfaces spread wider with significantly improved morphology. The cytoplasm extended outward, and their filopodia and lamellipodia closely adhered to the porous micro–nano structure framework on the material surfaces. Subsequently, RT-qPCR technology was used to detect the expression levels of six osteogenesis-related genes (*Alp, Bmp*, *Bsp, Opg*, *Opn* and *Runx2*) in BMSCs (Figure 5E–J). There was no significant difference in alkaline phosphatase (*Alp*) expression among the three groups of materials. For *Bmp*, the expression levels in the nHA-C/P group and the 5% Mn/nHA-C/P group were extremely significantly higher than those in the C/P group (*P *< 0.001). Regarding bone sialoprotein (*Bsp*), the expression level in the 5% Mn/nHA-C/P group was significantly different from that in the nHA-C/P group and the C/P group (*P *< 0.01). At the osteoprotegerin (*Opg*) level, the expression levels in the nHA-C/P group and the 5% Mn/nHA-C/P group were significantly upregulated compared with the C/P group (*P *< 0.01). For the osteopontin (*Opn*) gene, the expression levels in the nHA-C/P group and the 5% Mn/nHA-C/P group were extremely significantly higher than those in the C/P group (*P *< 0.001). Additionally, the expression levels of the runt-related transcription factor 2 (*Runx2*) gene in the nHA-C/P group and the 5% Mn/nHA-C/P group were significantly different from that in the C/P group (*P *< 0.05).

To evaluate the osteogenic differentiation potential of the materials, ALP staining was performed to analyze the osteogenic differentiation ability of cells on the material surfaces (Figure 5K). During the 1-, 4- and 7-day culture periods, the ALP staining of cells on C/P, nHA-C/P and 5% Mn/nHA-C/P surfaces gradually deepened with time. However, at the same time point, the C/P group showed the lightest staining, the nHA-C/P group showed increased staining intensity and the 5% Mn/nHA-C/P group showed the deepest staining. This indicated that the introduction of nHA enhanced the osteoinductivity of the material, and the doping of Mn further strengthened this effect, with 5% Mn/nHA-C/P exhibiting the optimal promoting effect on the osteogenic differentiation of cells.

### *In vivo* osseointegration evaluation of 5% Mn/nHA-C/P

**Figure 6:**
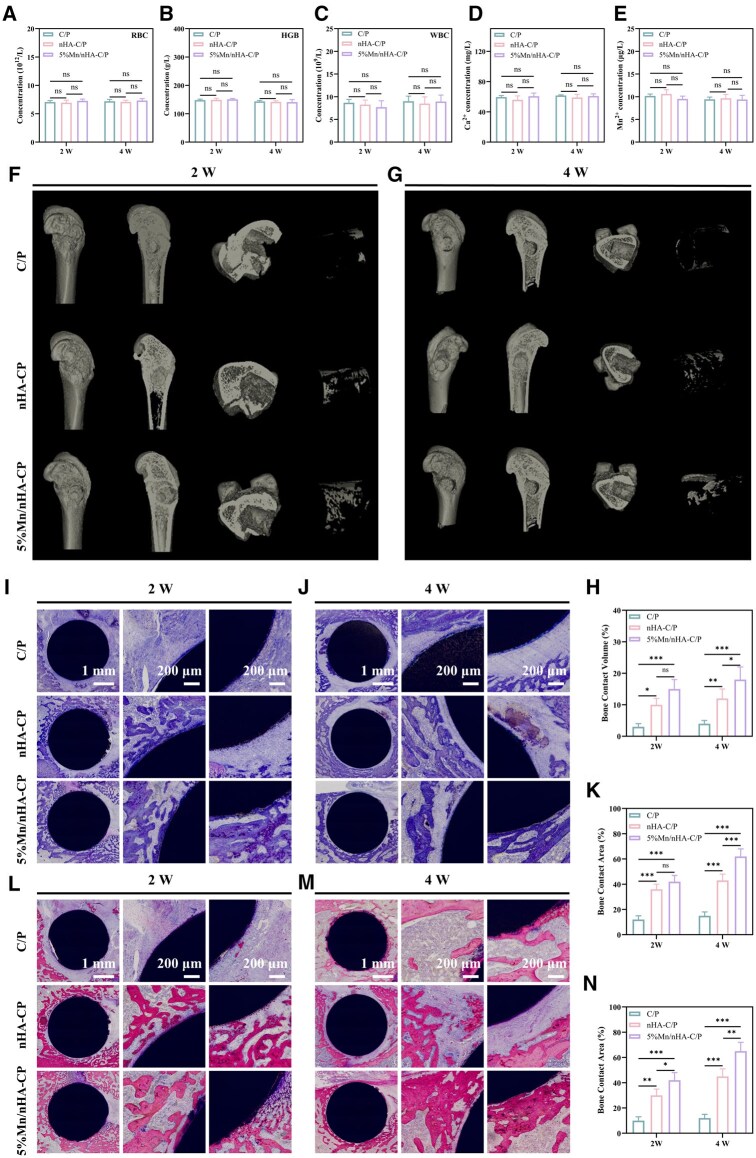
*In vivo* biocompatibility and osteogenic performance of C/P, nHA-C/P and 5% Mn/nHA-C/P scaffolds. (**A–E**) Serum levels of (**A**) RBC, (**B**) HGB, (**C**) WBC, (**D**) Ca^2+^ and (**E**) Mn^2+^ in rats at 2 weeks and 4 weeks after scaffold implantation. (**F, G**) μCT 3D reconstruction images of rat femoral condyle defects implanted with the three scaffolds at (**F**) 2 weeks and (**G**) 4 weeks. (**H**) Quantitative μCT analysis of bone contact volume. (**I, J**) Toluidine blue staining of defect sections at (**I**) 2 weeks and (**J**) 4 weeks; scale bars: 1 mm (left panels), 200 μm (middle/right panels). (**K**) Bone contact area of toluidine blue staining at 2 and 4 weeks. (**L, M**) Methylene blue-basic fuchsin staining of defect sections at (**L**) 2 weeks and (**M**) 4 weeks; scale bars: 1 mm (left panels), 200 μm (middle/right panels). (**N**) Bone contact area ratio of methylene blue-basic fuchsin staining at 2 weeks and 4 weeks. * refers to *P *< 0.05, ** refers to *P *< 0.01, *** refers to *P *< 0.001.

Following implantation for 2 weeks and 4 weeks, blood samples were collected. Routine blood test results (Figure 6A–C) showed that the levels of white blood cells, red blood cells and hemoglobin in the three groups were all within the normal range, indicating that all three materials have no obvious hematotoxicity and possess good biocompatibility. Meanwhile, the contents of calcium and Mn in the blood were detected (Figure 6D and E), and the results showed no significant differences among the three groups. To investigate the repair effect after material implantation, microcomputed tomography (μ-CT) was used for 3D reconstruction of bone tissue samples at 2 weeks and 4 weeks postoperatively (Figure 6F and G). Only a small amount of new bone contact was observed around the material in the C/P group, while the bone tissue contact volume in the nHA-C/P and 5% Mn/nHA-C/P groups was significantly higher. Moreover, with the extension of time, the amount of bone tissue around the two modified materials further increased, both higher than that in the C/P group. Quantitative analysis of bone tissue volume based on 3D reconstructed images (Figure 6H) revealed that at 2 weeks, the bone-to-implant contact volume fraction of the 5% Mn/nHA-C/P group reached 15%. While this value did not differ significantly from that of the nHA-C/P group, it was 5-fold higher than that of the C/P group. By 4 weeks, the bone-to-implant contact volume fractions increased across all the three groups compared to the 2-weeks time point, with the 5% Mn/nHA-C/P group still exhibiting a significant advantage, being 4.5-fold and 1.5-fold higher than those of the C/P and nHA-C/P groups, respectively.

Furthermore, histological samples were observed using toluidine blue staining and methylene blue-basic fuchsin staining (Figure 6I–L). It was found that a large amount of fibrous tissue surrounded the C/P group, forming a fibrous capsule, and the surrounding fibrous tissue did not decrease with prolonged implantation time. However, after coating modification, although some fibrous tissue was present around nHA-C/P and 5% Mn/nHA-C/P, these fibrous tissues gradually began to be replaced by mineralized bone tissue as the implantation time increased. Additionally, it was observed that compared to 2 weeks, the bone tissue surrounding the implants at 4 weeks exhibited deeper staining, indicating a gradual increase in mineralization. High-magnification images revealed a distinct gap between the C/P implants and the surrounding tissue, whereas no such gap was observed around nHA-C/P and 5% Mn/nHA-C/P implants.

Quantitative analysis of the bone contact area in the two staining sections was conducted to evaluate the osseointegration capability of different implants (Figure 6K–N). It was found that the bone contact area fraction in the C/P group showed almost no increase throughout the cultivation period, indicating that the implants were consistently surrounded by a large amount of fibrous tissue over the long term. In contrast, the bone contact area fractions in the nHA-C/P and 5% Mn/nHA-C/P groups increased significantly with prolonged implantation time, suggesting that the fibrous tissue around these implants was gradually being replaced by newly formed bone tissue. Notably, throughout the entire cultivation period, the two coating-modified implant groups, nHA-C/P and 5% Mn/nHA-C/P, exhibited significantly higher bone contact area fractions compared to C/P, with 5% Mn/nHA-C/P showing the highest contact area.

## Discussion

C/P composites exhibit excellent mechanical properties, biocompatibility and clinical radiolucency, making them promising substitutes for medical metals [5]. However, the inherent bioinertness can induce fibrosis at the implantation interface, significantly undermining the long-term clinical stability and repair efficacy of the C/P [6, 7]. A crucial factor underlying the interfacial fibrosis is the inability of material to actively regulate macrophage polarization phenotypes, leading to a predominant shift toward the pro-inflammatory M1 phenotype [37]. To address this issue, this study constructed a Mn/nHA coating on the surface of C/P through a liquid-phase nanoparticle self-assembly strategy, which endows the material with immunomodulatory and bone regeneration-promoting capabilities, thereby overcoming interfacial fibrosis upon implantation.

To achieve the *in situ* self-assembly of nanoparticles on the C/P surface, it is essential to modify the material to create nucleation sites [34, 38]. In this study, C/P was first pretreated through sulfonation to form a multi-microporous network and introduce hydrophilic nucleation sites (Figure 2). However, due to the presence of carbon fibers, the sulfonation treatment of C/P is more challenging compared to pure PEEK. Prolonged sulfonation may expose the carbon fibers within the PEEK matrix, increasing application risks, while short-time treatment tends to form an uneven network structure on the material surface. In this research, after appropriate sulfonation treatment of the C/P surface, the negatively charged sulfonic acid groups were further combined with positively charged monobutyl-calcium complexes to *in situ* form an MPN network rich in active groups and with strong adhesion. Utilizing the Ca^2+^ and abundant active groups in the MPN network, free Ca^2+^ and ${\mathrm{PO}}_{4}^{3-}$ ions in the solution were recruited onto the material surface, gradually growing from initially formed aggregates into complete HA nanoparticles. During this process, part of the Ca was replaced by Mn, and due to the matching ionic radii of Mn^2+^ and Ca^2+^, no significant morphological or phase distortion occurred (Figure 3). This solution-based strategy enables uniform and conformal coating deposition on implants of various sizes and complex geometries, ensuring consistent coverage and performance. Meanwhile, the coating exhibits excellent thermal and structural stability under standard autoclaving (121°C for 30 min), with no obvious changes in phase composition, elemental distribution, or morphology. These features ensure the reliability and practicality of the coating in clinical applications.

The uniform doping of Mn in nHA ensures the sustained regulation of macrophage polarization by the coating-modified C/P. Macrophage polarization is critical for regulating inflammatory responses and tissue repair, with distinct phenotypic marker genes playing well-defined roles [17]. CD206 (M2 surface marker) participates in injured tissue recognition, extracellular matrix remodeling and anti-inflammatory microenvironment establishment [39]; ARG (M2 intracellular gene) promotes collagen synthesis and tissue repair via arginine metabolism, a core indicator of M2 pro-repair function [40]. In contrast, CCR7 (M1 surface marker) mediates macrophage migration/recruitment to inflammatory sites, initiating early pro-inflammatory responses [41]; iNOS (M1 intracellular gene) exerts anti-microbial and pro-inflammatory effects by catalyzing nitric oxide production [42]. Therefore, *Cd206*/*Ccr7* and *Arg1*/*Nos2* ratios were used as comprehensive indicators to accurately reflect M1/M2 phenotypic transition and overall immune regulatory status (Figure 4). Compared with the pure C/P scaffold, both nHA-C/P and 5% Mn/nHA-C/P modified scaffolds more effectively activate the early immune inflammatory response. Notably, the 5% Mn/nHA-C/P scaffold, through the synergistic regulatory effect of Mn^2+^, more rapidly drives the phenotypic transition of macrophages from M1 to M2, enabling timely resolution of inflammation and rapid establishment of a reparative microenvironment [43]. Throughout this process, Mn^2+^ served as the key driver of this regulatory effect, achieving precise immune modulation through the synergistic action of direct and indirect dual pathways. In the direct pathway, Mn^2+^ activates signaling pathways such as NF-κB to regulate the transcriptional expression of macrophage functional markers [44], while in the indirect pathway, it synergizes with Ca^2+^ released from nHA to construct a specific ionic microenvironment that regulates the activity of surface receptors on immune cells [45].

Further *in vitro* osteogenic performance evaluation indicates that nHA can significantly enhance the adhesion, spreading and osteogenic differentiation capabilities of BMSCs, with Mn doping further amplifying this effect (Figure 5). Observations from cytoskeleton staining and SEM reveal that the microporous structure and nanocoatings on the surface of the modified materials substantially improve cell attachment efficiency. The adequate extension of filopodia and lamellipodia demonstrates the formation of stable bonds between cells and the material interface, which is closely associated with the increased surface roughness and improved hydrophilicity of the materials. Additionally, the sustained release and action of Mn/nHA provide an appropriate ionic microenvironment for the osteogenic differentiation of BMSCs. Previous studies have also reported that Ca^2+^ can promote the expression of osteogenic genes by modulating intracellular calcium signaling pathways [46, 47]. Moreover, Mn has been shown to activate the BMP/Smad signaling pathway, significantly upregulating the expression of *Runx2*, *Alp* and *Ocn*, thereby further enhancing the osteogenic differentiation of BMSCs [48].

Excellent immunomodulatory capability enables the Mn/nHA-modified C/P implant to initially clear foreign bodies in the injured area through high M1-type regulatory activity and activate local macrophage-mediated immune responses (Figure 6). Subsequently, it shifts the phenotype of surrounding macrophages toward the M2 type and leverages anti-inflammatory repair functions mediated by markers such as CD206 and ARG to create a microenvironment conducive to bone regeneration. In contrast, C/P without immunomodulatory function remains in a state of excessive immune activation after implantation, leading to rapid proliferation of fibroblasts and the formation of a fibrous capsule. Meanwhile, nHA-modified C/P, which only possesses single M1-type regulatory capacity, exhibits a significantly higher degree of fibrosis compared to Mn/nHA-modified materials. More importantly, the enhanced immunomodulatory capacity not only provides a direct regenerative microenvironment for bone tissue, but also promotes the migration of BMSCs to the defect site through secreted chemokines. Subsequently, by secreting a series of growth factors such as BMP2, VEGF and bFGF, it regulates the osteogenic-related responses of BMSCs and the functional expression of angiogenic cells, thereby coupling to achieve rapid osseointegration at the C/P implantation interface.

However, it should be noted that the observation period of the *in vivo*/*in vitro* experiments was relatively short (7 days for *in vitro* osteogenic assays and 4 weeks for *in vivo* experiments), because this study focuses on the immune-osteogenic coupling effect during bone regeneration, which primarily occurs and exerts its effects during the early stages of bone repair. Further studies with extended *in vitro* culture duration (14–21 days) and *in vivo* implantation time (8 weeks or longer) will be conducted in future investigations to comprehensively evaluate the long-term osteogenic performance and osseointegration of the Mn-doped composite.

## Conclusion

In this study, we constructed a Mn/nHA coating on the surface of C/P composite through a liquid-phase nanoparticle self-assembly strategy. This coating exhibits excellent immunomodulatory capability, which allows C/P to retain M1-type pro-inflammatory activity in the early implantation stage to activate local immunity, and later shifts to an M2-type anti-inflammatory repair phenotype to regulate the bone regeneration microenvironment. Meanwhile, the Mn/nHA-coated C/P also promotes the adhesion, proliferation and osteogenic differentiation of BMSCs. Ultimately, by coupling osteogenesis with immunomodulation, the strategy achieves rapid interfacial osseointegration of C/P in critical bone defects. This study provides new insights for the immunomodulation-osteogenesis synergistic design of bone repair materials.

## Supplementary Material

rbag085_Supplementary_Data

## References

[1] Yuan B , Peng H, Wang Y, Li J, Zhang Y, Chen Z, Li K, Tu C, Zhang K, Zhu X, Shen B, Nie Y, Zhang X. Micro/nanobiomimetic iron-based scaffold induces vascularized bone regeneration to repair large segmental bone defect in load-bearing sites. ACS Nano 2025;19:6840–57. 39933996 10.1021/acsnano.4c11960

[2] Shi S , Liao H, Lu W, Chen T, Sun Y, Lin Y. Stem cells recruited from multifunctional tetrahedral framework nucleic acids induce vascularized osteogenesis to repair bone defects. Adv Funct Materials 2025;35:1–15.

[3] Shuai Y , Yang T, Zheng M, Zheng L, Wang J, Mao C, Yang M. Oriented cortical-bone-like silk protein lamellae effectively repair large segmental bone defects in pigs. Adv Mater 2025;37:1–16. 10.1002/adma.202414543 PMC11899506 39871679

[4] Fan L , Chen S, Yang M, Liu Y, Liu J. Metallic materials for bone repair. Adv Healthc Mater 2024;13:e2302132. 37883735 10.1002/adhm.202302132

[5] Zhao W , Yu R, Dong W, Luan J, Wang G, Zhang H, Zhang M. The influence of long carbon fiber and its orientation on the properties of three-dimensional needle-punched CF/PEEK composites. Compos Sci Technol 2021;203:108565.

[6] Zhang Z , Zhang X, Zheng Z, Xin J, Han S, Qi J, Zhang T, Wang Y, Zhang S. Latest advances: improving the anti-inflammatory and immunomodulatory properties of PEEK materials. Mater Today Bio 2023;22:100748. 10.1016/j.mtbio.2023.100748 PMC10432209 37600350

[7] Wang X , Pan L, Zheng A, Cao L, Wen J, Su T, Zhang X, Huang Q, Jiang X. Multifunctionalized carbon-fiber-reinforced polyetheretherketone implant for rapid osseointegration under infected environment. Bioact Mater 2023;24:236–50. 36606257 10.1016/j.bioactmat.2022.12.016 PMC9803906

[8] Wu J , Deng J, Theocharidis G, Sarrafian TL, Griffiths LG, Bronson RT, Veves A, Chen J, Yuk H, Zhao X. Adhesive anti-fibrotic interfaces on diverse organs. Nature 2024;630:360–7. 38778109 10.1038/s41586-024-07426-9 PMC11168934

[9] Lee JH , Shin SJ, Lee JH, Knowles JC, Lee HH, Kim HW. Adaptive immunity of materials: implications for tissue healing and regeneration. Bioact Mater 2024;41:499–522. 39206299 10.1016/j.bioactmat.2024.07.027 PMC11350271

[10] Zhang X , Zhang Y, Liu Y. Fibroblast activation and heterogeneity in fibrotic disease. Nat Rev Nephrol 2025;21:613–32. 40537561 10.1038/s41581-025-00969-8

[11] Zheng Y , Gao A, Bai J, Liao Q, Wu Y, Zhang W, Guan M, Tong L, Geng D, Zhao X, Chu PK, Wang H. A programmed surface on polyetheretherketone for sequentially dictating osteoimmunomodulation and bone regeneration to achieve ameliorative osseointegration under osteoporotic conditions. Bioact Mater 2022;14:364–76. 35386814 10.1016/j.bioactmat.2022.01.042 PMC8964985

[12] Zheng Y , Zhou H, Li M, Fu J, Dong J, Liu Y, Liu L. Polyetheretherketone surface engineered with a degradable hybrid coating for accelerating osteogenesis. Mater Lett 2023;331:133515.

[13] Chen L , Guo Z, Duan G, Zhao G, Liu X, Wang Z, Liu Y, Bai L, Xu H, Peng Y, Qiang L, Chang D, Sheng J, Sun T, Weng J. The multifaceted biomimetic titanium implant promotes bone integration with sequential antibacterial and immune modulation properties. Bioact Mater 2025;51:494–511. 40496628 10.1016/j.bioactmat.2025.05.015 PMC12149584

[14] Huang B , Li S, Dai S, Lu X, Wang P, Li X, Zhao Z, Wang Q, Li N, Wen J, Liu Y, Wang X, Man Z, Li W, Liu B. Ti3C2Tx MXene-decorated 3D-printed ceramic scaffolds for enhancing osteogenesis by spatiotemporally orchestrating inflammatory and bone repair responses. Adv Sci 2024;11:1–16. 10.1002/advs.202400229 PMC11425883 38973266

[15] Gong X , Wang S, Zhang J, Ma H, Sun Q, Yao M, Chen J, Zhang J, Huang Q, Shi X, Qi W, Lin Y, You B, Bai Y, Zhang G, Ai K. Tannin-mediated ischemia-homing-angiogenesis nanodots (IHAND) for synergistic angiogenesis and heart failure prevention post-myocardial infarction. Adv Mater 2025;14662:1–22. 10.1002/adma.202514662 41208520

[16] Liu Z , Song J, Heng BC, Wang Y, Xu M, He Y, Liu Y, Deng X, Zhang X. Magnetoelectric coupling stimulation modulates macrophage reprogramming for superior infected periodontal tissue regeneration. Adv Mater 2026;38:e2517222. 10.1002/adma.202517222 41479270

[17] Liu J , Chen F, Tian L, Wu J, Liu K, Wan Q, Yuan B, Zhu X, Chen X, Zhang X. Therapeutic potential of gelatine methacrylate hydrogels loaded with macrophage-derived exosomes for accelerating angiogenesis and cutaneous wound healing. Collagen Leather 2024;6:71–92.

[18] Cui S , Wu X, Yu X, Huang Y, Zhang D, Chen H, Heng BC, Cai Q, Wang Y, Gu Y, Deng X. SiAkt2-loaded nanoparticles reprogramming macrophages to M2 phenotype for effective bone defect repair. Adv Mater 2025;10507:1–15. 10.1002/adma.202410507 41189396

[19] You Y , Wei S, Gao Z, Wang L, Cheng Q, Chang M, Ma Q, Wang L, Hu X, Liu X, Cui J. Engineered poly(ethylene glycol)-alendronate-magnesium hydrogels potentiate site-specific immunomodulation for the healing of osteoporosis fractures. Adv Funct Mater 2025;22720:1–14.

[20] Li Y , Yu JL, Zhu J, Xiang F, Wang Y, Wang X, Liu J. C. Bioactive materials-mediated regulation of bone marrow microenvironment: mechanistic insights and therapeutic potentials. Adv Mater 2025;11497:1–29. 10.1002/adma.202511497 41031919

[21] Zhao R , Meng X, Pan Z, Li Y, Qian H, Zhu X, Yang X, Zhang X. Advancements in nanohydroxyapatite: synthesis, biomedical applications and composite developments. Regen Biomater 2025;12:rbae129. 39776858 10.1093/rb/rbae129 PMC11703556

[22] Qin D , Zhao Y, Cheng R, Liu Y, Guo S, Sun L, Guo Y, Hao F, Zhao B. Mussel-inspired immunomodulatory and osteoinductive dual-functional hydroxyapatite nanoplatform for promoting bone regeneration. J Nanobiotechnology 2024;22:320 38849820 10.1186/s12951-024-02593-3 PMC11162024

[23] Yang Y , Cui Y, Cao W, Zhao M, Lin W, Xu R, Xu Y, Chen Y, Li H, Liang J, Lin Y, Fan Y, Zhang X, Sun Y. Nanohydroxyapatite stimulates PD-L1 expression to boost melanoma combination immunotherapy. ACS Nano 2022;16:18921–35. 36315589 10.1021/acsnano.2c07818

[24] Zeng Q , Wang R, Hua Y, Wu H, Chen X, Xiao YC, Ao Q, Zhu X, Zhang X. Hydroxyapatite nanoparticles drive the potency of toll-like receptor 9 agonist for amplified innate and adaptive immune response. Nano Res 2022;15:9286–97. 35911480 10.1007/s12274-022-4683-x PMC9308403

[25] Li J , Deng C, Liang W, Kang F, Bai Y, Ma B, Wu C, Dong S. Mn-containing bioceramics inhibit osteoclastogenesis and promote osteoporotic bone regeneration via scavenging ROS. Bioact Mater 2021;6:3839–50. 33898880 10.1016/j.bioactmat.2021.03.039 PMC8050801

[26] Zheng Y , Chen J, Song XR, Chang MQ, Feng W, Huang H, Jia CX, Ding L, Chen Y, Wu R. Manganese-enriched photonic/catalytic nanomedicine augments synergistic anti-TNBC photothermal/nanocatalytic/immuno-therapy via activating CGAS-STING pathway. Biomaterials 2023;293:121988. 36580716 10.1016/j.biomaterials.2022.121988

[27] Wang C , Guan Y, Lv M, Zhang R, Guo Z, Wei X, Du X, Yang J, Li T, Wan Y, Su X, Huang X, Jiang Z. Manganese increases the sensitivity of the CGAS-STING pathway for double-stranded DNA and is required for the host defense against DNA viruses. Immunity 2018;48:675–87.e7. 29653696 10.1016/j.immuni.2018.03.017

[28] Abdulhussein HJ , Mohsin MH, Jabir MS, Sulaiman GM, Mohammed HA, Ismail RA, Ramizy A, Eisa MH, Ibrahim NA, Badher NS. Eco-friendly synthesis of eggshell-derived nano-hydroxyapatite: physicochemical characterization, hemocompatibility, and bone regeneration potential. Sci Rep 2025;15:32832. 40998943 10.1038/s41598-025-17486-0 PMC12464236

[29] Wu H , Wang R, Li S, Chen S, Liu S, Li X, Yang X, Zeng Q, Zhou Y, Zhu X, Zhang K, Tu C, Zhang X. Aspect ratio-dependent dual-regulation of the tumor immune microenvironment against osteosarcoma by hydroxyapatite nanoparticles. Acta Biomater 2023;170:427–41. 37634831 10.1016/j.actbio.2023.08.046

[30] Wang Y , Wu H, Chen Z, Cao J, Zhu X, Zhang X. Nano-hydroxyapatite promotes cell apoptosis by co-activating endoplasmic reticulum stress and mitochondria damage to inhibit glioma growth. Regen Biomater 2024;11:rbae038. 38799701 10.1093/rb/rbae038 PMC11127112

[31] Wu H , Hua Y, Wu J, Zeng Q, Yang X, Zhu X, Zhang X. The morphology of hydroxyapatite nanoparticles regulates clathrin-mediated endocytosis in melanoma cells and resultant anti-tumor efficiency. Nano Res 2022;15:6256–65.

[32] Cai L , Han F, Ding J, Zhou X, Shi T, Cheng F, Peng C, Long S, Sun W, Fan J, Du J, Peng X. Biodegradable and piezoelectric Mn-doped hydroxyapatite for sonodynamic immunotherapy. ACS Nano 2025;19:24067–77. 40554728 10.1021/acsnano.5c06775

[33] Xu P , Gu Y, Li C, Shen J, Cheng X, Zhang LW, Wang Y, Wang Y. Radioactive hydroxyapatite microspheres empower sustainable in situ tumor vaccination. ACS Nano 2024;18:18425–43. 38975713 10.1021/acsnano.4c02972

[34] Lee S , Chang YY, Lee J, Madhurakkat Perikamana SK, Kim EM, Jung YH, Yun JH, Shin H. Surface engineering of titanium alloy using metal–polyphenol network coating with magnesium ions for improved osseointegration. Biomater Sci 2020;8:3404–17. 32377652 10.1039/d0bm00566e

[35] Li Y , Nam CT, Ooi CP. Iron(III) and manganese(II) substituted hydroxyapatite nanoparticles: characterization and cytotoxicity analysis. J Phys: Conf Ser 2009;187:012024.

[36] Kaviya M , Ramakrishnan P, Mohamed SB, Ramakrishnan R, Gimbun J, Veerabadran KM, Kuppusamy MR, Kaviyarasu K, Sridhar TM. Synthesis and characterization of nano-hydroxyapatite/graphene oxide composite materials for medical implant coating applications. Mater Today Proc 2021;36:204–7.

[37] Witherel CE , Abebayehu D, Barker TH, Spiller KL. 16 Fbr. Adv Healthc Mater 2019;8:1–35. 10.1002/adhm.201801451 PMC6415913 30658015

[38] Tang M , Wang Y, Yang X, Xue J, Wang L, Wang S, Li J, Song C, Lu H, Pang X, Chen B, Zhang Y. The synergistic effects of modified carbon fiber and multiwalled carbon nanotubes on the tribological properties of polyetheretherketone composites. ACS Appl Polym Mater 2025;7:11248–61.

[39] Wang J , Xu L, Xiang Z, Ren Y, Zheng X, Zhao Q, Zhou Q, Zhou Y, Xu L, Wang Y. Microcystin-LR ameliorates pulmonary fibrosis via modulating CD206+ M2-like macrophage polarization. Cell Death Dis 2020;11:136. 32075954 10.1038/s41419-020-2329-z PMC7031231

[40] Yang Z , Ming XF. Functions of arginase isoforms in macrophage inflammatory responses: impact on cardiovascular diseases and metabolic disorders. Front Immunol 2014;5:533. 25386179 10.3389/fimmu.2014.00533 PMC4209887

[41] Xuan W , Qu Q, Zheng B, Xiong S, Fan G-H. The chemotaxis of M1 and M2 macrophages is regulated by different chemokines. J Leukoc Biol 2015;97:61–9. 25359998 10.1189/jlb.1A0314-170R

[42] Palmieri EM , Gonzalez-Cotto M, Baseler WA, Davies LC, Ghesquière B, Maio N, Rice CM, Rouault TA, Cassel T, Higashi RM, Lane AN, Fan TWM, Wink DA, McVicar DW. Nitric oxide orchestrates metabolic rewiring in M1 macrophages by targeting aconitase 2 and pyruvate dehydrogenase. Nat Commun 2020;11:698. 32019928 10.1038/s41467-020-14433-7 PMC7000728

[43] Wang D , Wang Y, Zhang Q, Xi W, Lu Y, Wang S, Zheng L, Kang Y, Chi X. A shikonin-based anti-glycolysis platform orchestrating macrophage activation for sepsis therapy. Chem Eng J 2025;526:171056.

[44] Chen H , Wang D, Liu J, Chen J, Hu Y, Ni Y. Augmenting antitumor immune effects through the coactivation of CGAS-STING and NF-ΚB crosstalk in dendritic cells and macrophages by engineered manganese ferrite nanohybrids. ACS Appl Mater Interfaces 2025;17:13375–90. 39964151 10.1021/acsami.4c18570

[45] Li R , Zhu Z, Zhang B, Jiang T, Zhu C, Mei P, Wang JY, Li R, Guo Y, Liu W, Xia C, Fang LB. Manganese enhances the osteogenic effect of silicon-hydroxyapatite nanowires by targeting T lymphocyte polarization. Adv Sci 2024;11:1–18. 10.1002/advs.202305890 PMC10811488 38039434

[46] Kito H , Ohya S. Role of K+ and Ca2+-permeable channels in osteoblast functions. Int J Mol Sci 2021;22:10459. 34638799 10.3390/ijms221910459 PMC8509041

[47] Wang Y , Pan J, Zhang Y, Li X, Zhang Z, Wang P, Qin Z, Li J. Wnt and notch signaling pathways in calcium phosphate-enhanced osteogenic differentiation: a pilot study. J Biomed Mater Res B Appl Biomater 2019;107:149–60. 29569393 10.1002/jbm.b.34105

[48] Awais M , Aizaz A, Nazneen A, Bhatti Q ul A, Akhtar M, Wadood A, Atiq Ur Rehman M. A review on the recent advancements on therapeutic effects of ions in the physiological environments. Prosthesis 2022;4:263–316.

[49] Muthusamy S , Mahendiran B, Sampath S, Jaisankar SN, Anandasadagopan SK, Krishnakumar GS. Hydroxyapatite nanophases augmented with selenium and manganese ions for bone regeneration: physiochemical, microstructural and biological characterization. Mater Sci Eng C Mater Biol Appl 2021;126:112149. 34082960 10.1016/j.msec.2021.112149

