# Supplementary material for: Manganese-doped nano-hydroxyapatite enhances CF/PEEK osseointegration via immunomodulation-osteogenesis coupling
Source: Regen Biomater. 2026 May 4;13:rbag085. doi: 10.1093/rb/rbag085 (PMC13211982; doi:10.1093/rb/rbag085)
Supplement: rbag085_Supplementary_Data [file rbag085_supplementary_data.docx]

**Manganese-doped nano-hydroxyapatite enhances osseointegration of CF/PEEK osseointegration via immunomodulation-osteogenesis coupling**

*Jiajun Liu ^1,2^, Zhenghao Li ^1,2^, Xuening Chen ^1,2^, Kai Zhang^1,2^, Xiangdong Zhu ^1,2^, Bo Yuan^1,2,*^*

^1^ National Engineering Research Center for Biomaterials, Sichuan University, Chengdu, 610064, China

^2^ College of Biomedical Engineering, Sichuan University, Chengdu, 610064, China

*Corresponding authors: Bo Yuan (scuyb@scu.edu.cn)

**Supporting figures and tables**


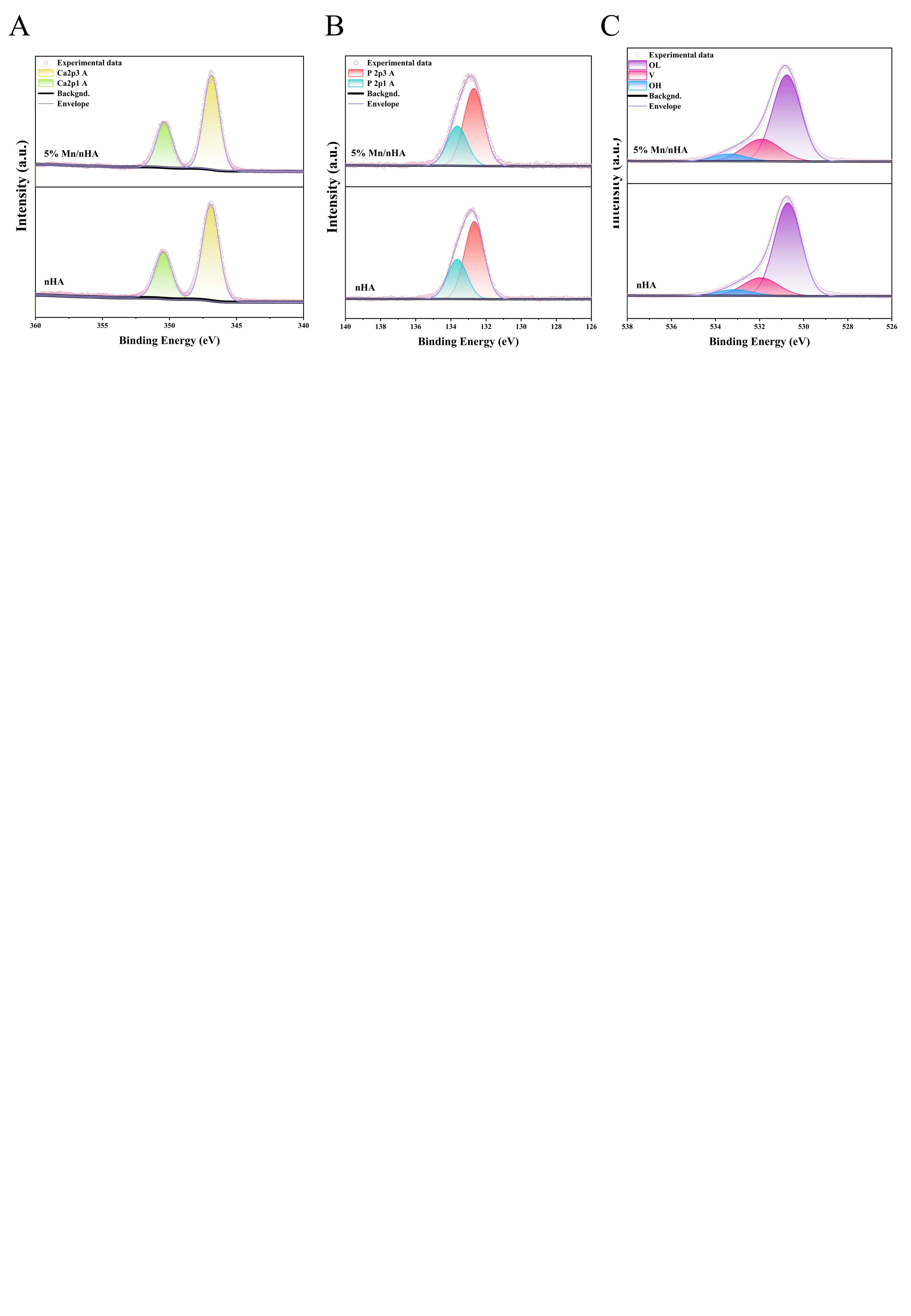


Tables S1 Primer sequence used in RT-qPCR analysis.

| Name | Sequence（5’-3’） |
| --- | --- |
| *Ccr7* | F: GGAAACCCAGGAAAAACGTGC |
|  | R: CCGTGGTATTCTCGCCGATG |
| *Nos2* | F: CATTGGAAGTGAAGCGTTTCG |
|  | R: CAGCTGGGCTGTACAAACCTT |
| *Cd206* | F: GCACTGGGTTGCATTGGTTT |
|  | R: TGCAGGGTTGACATGAGACC |
| *Arg1* | F: GAACACGGCAGTGGCTTTAAC |
|  | R: TGCTTAGCTCTGTCTGCTTTGC |
| *mGAPDH* | F: ACCCAGAAGACTGTGGATGG |
|  | R: CACATTGGGGGTAGGAACAC |
| *Alp* | F: CATCGCCTATCAGCTAATGCACA |
|  | R: ATGAGGTCCAGGCCATCCAG |
| *Bmp* | F: TGACTGGATCGTGGCACCTC |
|  | R: CAGAGTCTGCACTATGGCATGGTTA |
| *Bsp* | F: GACCAGTTATGGCACCACGA |
|  | R: CGCAGTGTTGTACTCGTTGC |
| *Opg* | F: AACCGCACCCACAACCGA |
|  | R: CACCTGAGAAGAACCCATCCG |
| *Opn* | F: GCAGAATCTTCTAGCCCCACA |
|  | R: CATGGTCTCCGTCGTCATCG |
| *Runx2* | F: TGGCCGGGAATGATGAGAAC |
|  | R: TTGAACCTGGCCACTTGGTT |
| *rGAPDH* | F: GGCACAGTCAAGGCTGAGAATG |
|  | R: ATGGTGGTGAAGACGCCAGTA |
